# Supplementary material for: Resolving the full spectrum of human genome variation using Linked-Reads
Source: Genome Res. 2019 Apr;29(4):635–45. doi: 10.1101/gr.234443.118 (PMC6442396; doi:10.1101/gr.234443.118)
Supplement: Supplemental Material [file supp_gr.234443.118_Supplemental_longranger-2.2.zip › longranger-2.2.tar.bz2!/lr2.2-public/lib/stan/cmdstan/stan/doc/api/html/to__fvar_8hpp_source.html]

Stan: src/stan/agrad/fwd/matrix/to\_fvar.hpp Source File


|  |  |
| --- | --- |
|  | Stan  2.5.0  probability, sampling & optimization |


- Main Page
- Related Pages
- Namespaces
- Classes
- Files

- File List
- File Members

All Classes Namespaces Files Functions Variables Typedefs Enumerations Enumerator Friends Macros Pages

- src
- stan
- agrad
- fwd
- matrix

to\_fvar.hpp

Go to the documentation of this file.

1 #ifndef STAN\_\_AGRAD\_\_FWD\_\_MATRIX\_\_TO\_FVAR\_HPP

2 #define STAN\_\_AGRAD\_\_FWD\_\_MATRIX\_\_TO\_FVAR\_HPP

3

4 #include <vector>

5 #include <stan/math/matrix/Eigen.hpp>

6 #include <stan/math/matrix/typedefs.hpp>

7 #include <stan/agrad/fwd/fvar.hpp>

8 #include <stan/agrad/fwd/matrix/typedefs.hpp>

9 #include <stan/math/error\_handling/matrix/check\_matching\_dims.hpp>

10

11 namespace stan {

12  namespace agrad {

13

14  template<typename T>

15  inline

16  fvar<T>

17  to\_fvar(const T& x) {

18  return fvar<T>(x);

19  }

20

21  template<typename T>

22  inline

23  fvar<T>

24  to\_fvar(const fvar<T>& x) {

25  return x;

26  }

27

28  inline

29  matrix\_fd

30  to\_fvar(const stan::math::matrix\_d& m) {

31  matrix\_fd m\_v(m.rows(), m.cols());

32  for (int j = 0; j < m.cols(); ++j)

33  for (int i = 0; i < m.rows(); ++i)

34  m\_v(i,j) = m(i,j);

35  return m\_v;

36  }

37

38  inline

39  matrix\_fd

40  to\_fvar(const matrix\_fd& m) {

41  return m;

42  }

43

44  inline

45  matrix\_fv

46  to\_fvar(const matrix\_fv& m) {

47  return m;

48  }

49

50  inline

51  matrix\_ffd

52  to\_fvar(const matrix\_ffd& m) {

53  return m;

54  }

55

56  inline

57  matrix\_ffv

58  to\_fvar(const matrix\_ffv& m) {

59  return m;

60  }

61

62  inline

63  vector\_fd

64  to\_fvar(const stan::math::vector\_d& v) {

65  vector\_fd v\_v(v.size());

66  for (int i = 0; i < v.size(); ++i)

67  v\_v[i] = v[i];

68  return v\_v;

69  }

70

71  inline

72  vector\_fd

73  to\_fvar(const vector\_fd& v) {

74  return v;

75  }

76

77  inline

78  vector\_fv

79  to\_fvar(const vector\_fv& v) {

80  return v;

81  }

82

83  inline

84  vector\_ffd

85  to\_fvar(const vector\_ffd& v) {

86  return v;

87  }

88

89  inline

90  vector\_ffv

91  to\_fvar(const vector\_ffv& v) {

92  return v;

93  }

94

95  inline

96  row\_vector\_fd

97  to\_fvar(const stan::math::row\_vector\_d& rv) {

98  row\_vector\_fd rv\_v(rv.size());

99  for (int i = 0; i < rv.size(); ++i)

100  rv\_v[i] = rv[i];

101  return rv\_v;

102  }

103

104  inline

105  row\_vector\_fd

106  to\_fvar(const row\_vector\_fd& rv) {

107  return rv;

108  }

109

110  inline

111  row\_vector\_fv

112  to\_fvar(const row\_vector\_fv& rv) {

113  return rv;

114  }

115

116  inline

117  row\_vector\_ffd

118  to\_fvar(const row\_vector\_ffd& rv) {

119  return rv;

120  }

121

122  inline

123  row\_vector\_ffv

124  to\_fvar(const row\_vector\_ffv& rv) {

125  return rv;

126  }

127

128  template<typename T, int R, int C>

129  inline

130  Eigen::Matrix<fvar<T>, R, C>

131  to\_fvar(const Eigen::Matrix<T,R,C>& val,

132  const Eigen::Matrix<T,R,C>& deriv) {

133

134  stan::math::check\_matching\_dims("to\_fvar(%1%)",val,"value",

135  deriv, "deriv",(double\*)0);

136  Eigen::Matrix<fvar<T>,R,C> ret(val.rows(), val.cols());

137  for(size\_type i = 0; i < val.rows(); i++) {

138  for(size\_type j = 0; j < val.cols(); j++) {

139  ret(i,j).val\_ = val(i,j);

140  ret(i,j).d\_ = deriv(i,j);

141  }

142  }

143  return ret;

144  }

145  }

146 }

147 #endif

check\_matching\_dims.hpp

typedefs.hpp

stan::agrad::row\_vector\_fd

Eigen::Matrix< fvar< double >, 1, Eigen::Dynamic > row\_vector\_fd

**Definition:** typedefs.hpp:50

stan::agrad::fvar

**Definition:** fvar.hpp:13

stan::agrad::to\_fvar

fvar< T > to\_fvar(const T &x)

**Definition:** to\_fvar.hpp:17

typedefs.hpp

stan::agrad::row\_vector\_ffv

Eigen::Matrix< fvar< fvar< var > >, 1, Eigen::Dynamic > row\_vector\_ffv

**Definition:** typedefs.hpp:62

stan::agrad::row\_vector\_fv

Eigen::Matrix< fvar< var >, 1, Eigen::Dynamic > row\_vector\_fv

**Definition:** typedefs.hpp:54

stan::math::check\_matching\_dims

bool check\_matching\_dims(const char \*function, const Eigen::Matrix< T1, R1, C1 > &y1, const char \*name1, const Eigen::Matrix< T2, R2, C2 > &y2, const char \*name2, T\_result \*result)

**Definition:** check\_matching\_dims.hpp:15

stan::agrad::size\_type

Eigen::Matrix< double, Eigen::Dynamic, Eigen::Dynamic >::Index size\_type

**Definition:** typedefs.hpp:14

stan::math::matrix\_d

Eigen::Matrix< double, Eigen::Dynamic, Eigen::Dynamic > matrix\_d

Type for matrix of double values.

**Definition:** typedefs.hpp:23

stan::agrad::vector\_fd

Eigen::Matrix< fvar< double >, Eigen::Dynamic, 1 > vector\_fd

**Definition:** typedefs.hpp:34

stan::agrad::row\_vector\_ffd

Eigen::Matrix< fvar< fvar< double > >, 1, Eigen::Dynamic > row\_vector\_ffd

**Definition:** typedefs.hpp:58

fvar.hpp

Eigen.hpp

stan::agrad::matrix\_fd

Eigen::Matrix< fvar< double >, Eigen::Dynamic, Eigen::Dynamic > matrix\_fd

**Definition:** typedefs.hpp:18

stan::agrad::matrix\_ffv

Eigen::Matrix< fvar< fvar< var > >, Eigen::Dynamic, Eigen::Dynamic > matrix\_ffv

**Definition:** typedefs.hpp:30

stan::agrad::vector\_fv

Eigen::Matrix< fvar< var >, Eigen::Dynamic, 1 > vector\_fv

**Definition:** typedefs.hpp:38

stan::agrad::matrix\_fv

Eigen::Matrix< fvar< var >, Eigen::Dynamic, Eigen::Dynamic > matrix\_fv

**Definition:** typedefs.hpp:22

stan::agrad::vector\_ffd

Eigen::Matrix< fvar< fvar< double > >, Eigen::Dynamic, 1 > vector\_ffd

**Definition:** typedefs.hpp:42

stan::math::vector\_d

Eigen::Matrix< double, Eigen::Dynamic, 1 > vector\_d

Type for (column) vector of double values.

**Definition:** typedefs.hpp:30

stan::agrad::vector\_ffv

Eigen::Matrix< fvar< fvar< var > >, Eigen::Dynamic, 1 > vector\_ffv

**Definition:** typedefs.hpp:46

stan::agrad::matrix\_ffd

Eigen::Matrix< fvar< fvar< double > >, Eigen::Dynamic, Eigen::Dynamic > matrix\_ffd

**Definition:** typedefs.hpp:26

stan::math::row\_vector\_d

Eigen::Matrix< double, 1, Eigen::Dynamic > row\_vector\_d

Type for (row) vector of double values.

**Definition:** typedefs.hpp:37


---

[ Stan Home Page ]

*© 2011–2014,
Stan Development Team.*
